# Regulation of Akt/FoxO3a/Skp2 Axis Is Critically Involved in Berberine-Induced Cell Cycle Arrest in Hepatocellular Carcinoma Cells

**DOI:** 10.3390/ijms19020327

**Published:** 2018-01-23

**Authors:** Fanni Li, Xiwen Dong, Peng Lin, Jianli Jiang

**Affiliations:** State Key Laboratory of Cancer Biology, Cell Engineering Research Center & Department of Cell Biology, Fourth Military Medical University, 169 Changle West Road, Xi’an 710032, China; fannycpu@163.com (F.L.); dongxiwen@fmmu.edu.cn (X.D.); linpengxtal@126.com (P.L.)

**Keywords:** hepatocellular carcinoma, berberine, Akt, FoxO3a, Skp2

## Abstract

The maintenance of ordinal cell cycle phases is a critical biological process in cancer genesis, which is a crucial target for anti-cancer drugs. As an important natural isoquinoline alkaloid from Chinese herbal medicine, Berberine (BBR) has been reported to possess anti-cancer potentiality to induce cell cycle arrest in hepatocellular carcinoma cells (HCC). However, the underlying mechanism remains to be elucidated. In our present study, G0/G1 phase cell cycle arrest was observed in berberine-treated Huh-7 and HepG2 cells. Mechanically, we observed that BBR could deactivate the Akt pathway, which consequently suppressed the S-phase kinase-associated protein 2 (Skp2) expression and enhanced the expression and translocation of Forkhead box O3a (FoxO3a) into nucleus. The translocated FoxO3a on one hand could directly promote the transcription of cyclin-dependent kinase inhibitors (CDKIs) p21^Cip1^ and p27^Kip1^, on the other hand, it could repress Skp2 expression, both of which lead to up-regulation of p21^Cip1^ and p27^Kip1^, causing G0/G1 phase cell cycle arrest in HCC. In conclusion, BBR promotes the expression of CDKIs p21^Cip1^ and p27^Kip1^ via regulating the Akt/FoxO3a/Skp2 axis and further induces HCC G0/G1 phase cell cycle arrest. This research uncovered a new mechanism of an anti-cancer effect of BBR.

## 1. Introduction

Hepatocellular carcinoma (HCC) is one of the most prevalent malignancies throughout the world, with a particularly high incidence in the Asian population. Despite the substantial advances in drug discovery and development, the treatment options for HCC are still limited [1,2]. Although chemotherapy and radiotherapy for HCC have significantly increased the survival rate, HCC treatment is still struggling due to considerable limitations and deleterious clinical side effects [3,4]. Herein, to develop safer effective agents in HCC therapy is urgently needed. Among all the therapeutic strategies, traditional Chinese medicine (TCM) and its phytochemicals have been reported to be effective in the treatment and improvement of quality of life (QOL) for HCC patients [5].Therefore, research to uncover the underlying mechanisms of the anti-tumor effect of TCM in HCC seems rather imperative.

Accumulated evidence has emerged, illuminating anti-cancer effects of phytochemical-berberine (BBR) [6], which is an isoquinoline type of botanical alkaloid present in many traditional Chinese medicines as the major bioactive compound. BBR has several potent pharmacological functions and has been used for its antidiabetic, antimicrobial, antidiabetic, antidiarrheal and anti-inflammatory activities [7,8,9]. Additionally, BBR has been reported to possess anti-tumor activity in multiple types of cancers including HCC [10,11]. According to previous studies, BBR could not only induce HCC apoptosis by regulating the p38 mitogen activated protein kinase (MAPK) pathway and reactive oxygen species (ROS) generation [12], but also inhibits HCC migration, invasion and metastasis through the induction of plasminogen activator inhibitor-1 (PAI-1) [13]. To the best of our knowledge, few studies have demonstrated, in detail, the underlying mechanism by which BBR could influence HCC cell cycle, a critical cellular activity concerning cancer cell proliferation and neoplastic transformation.

FoxO3a belongs to Forkhead box Type O (FoxO) family of transcription factors that are characterized by a distinct forkhead DNA-binding domain. Many studies have demonstrated that FoxO3a exerts tumor suppressive activities by negatively regulating cell proliferation, promoting cell cycle arrest, repairing damaged DNA and inducing cell apoptosis after translocating into nucleus [14]. Furthermore, the loss of FoxO3a could induce the epithelial-mesenchymal transition (EMT) and subsequently promote tumor cells metastasis, which enables FoxO3a to be a candidate marker for tumor metastasis [15]. Nevertheless, BBR could reduce FoxO3a phosphorylation, causing its translocation into the nucleus and subsequent regulation of cell cycle arrest [16]. Therefore, FoxO3a might be a tangible participant of BBR-induced cell cycle arrest [17].

Skp2 is a specific substrate-recognition subunit of the Skp1 Cullin-F-box protein (SCF) type ubiquitin ligase complex [18]. Skp2 has been reported to be up-regulated in a large number of human cancers, including prostate cancer, breast cancer, lung cancer, colorectal carcinoma, liver cancer and others [19].The Skp2-containning SCF (SCF^Skp2^) has been previously reported to participate in proteasomal degradation of cyclin-dependent kinase inhibitors (CDKIs) that are key regulators of cell cycle [20]. Therefore, to investigate the mechanism by which BBR influences HCC cell cycle, we carried out the research to discover potential relationships among FoxO3a, Skp2 and CKDIs. The consequent findings can provide new insights on how BBR inhibits tumor proliferation and exerts anti-cancer activity.

## 2. Results

### 2.1. BBR Inhibits the Proliferation of Huh-7 and HepG2 Cells

**Figure 1:**
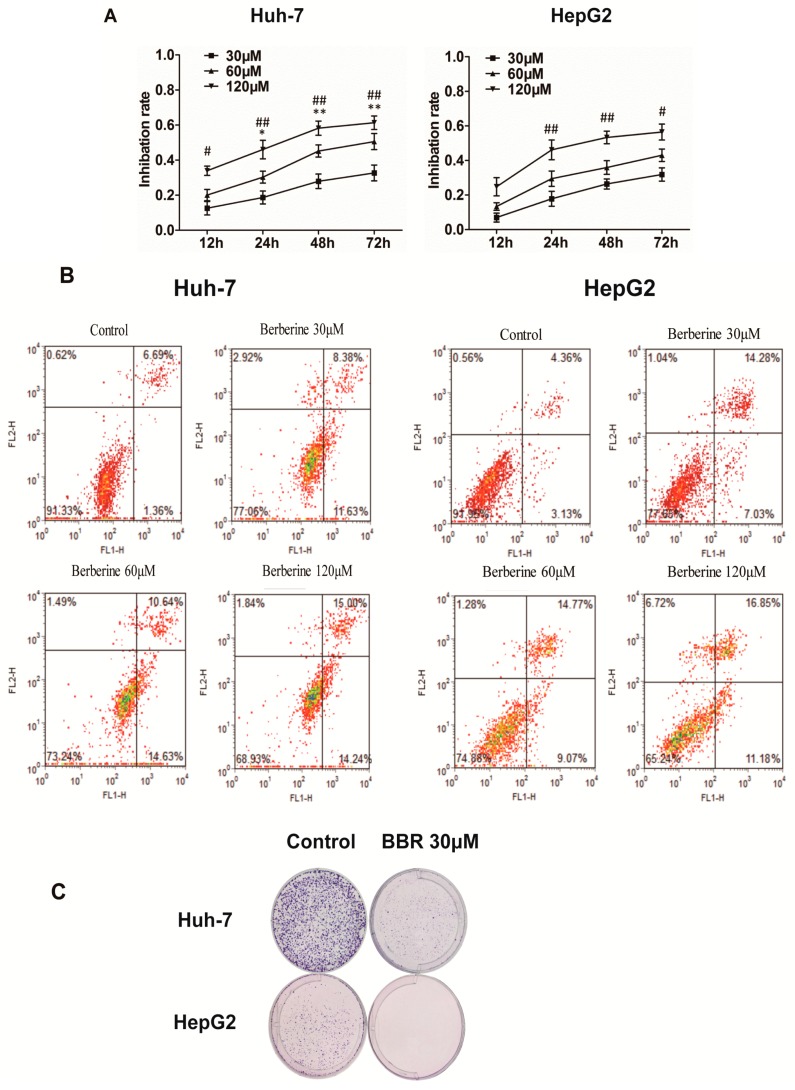
Berberine treatment inhibits the viability and clonogenicity of Huh-7 and HepG2 cells. (**A**) Inhibition rate of indicated cells after berberine (30–120 μM) treatment for 12–72 h was detected by CCK8 assay. In the line graphs, * represents *p* < 0.05, and ** represents *p* < 0.01 (60 vs. 30 μM); # represents *p* < 0.05, and ## represents *p* < 0.01 (120 vs. 30 μM). The experiments were carried out for three times; (**B**) Flow cytometry analysis of apoptosis cells assessed using annexin V/PI dual staining after 24 h treatment of Huh-7 and HepG2 cells with 30, 60, and 120 μM berberine; (**C**) Clonogenic analysis of Huh-7 and HepG2 cells after berberine treatment. The indicated cells were cultured with DMSO (control) or 30 μM berberine for 14 days. Colonies were fixed and stained with Giemsa solution. PI: propidium iodide; BBR: berberine.

The cell counting kit-8 (CCK8) assay was performed to detect the effect of BBR on the viability (under concentrations of 0, 30, 60 and 120 μM for 12–72 h) of Huh-7 and HepG2 cells. The results demonstrated that Huh-7 cells were more sensitive to alteration of BBR concentration, for the increased degree of inhibition were significant between 30 and 60 μM of BBR after 24 h treatment (1.6 fold at 24 h, *p* < 0.05; 1.65-fold at 48 h, *p* < 0.01; and 1.56 fold at 72 h, *p* < 0.01), while those were minute of HepG2 cells (Figure 1A). However, HepG2 cells were more rapid-changing as inhibition rate increased more within 24 h (17% for 30 μM, 28% for 60 μM and 45% for 120 μM) than those in 24–72 h (14% for 30 μM, 14% for 60 μM and 11% for 120 μM). Additionally, the apoptosis-inducing potential of BBR was confirmed using annexin V/propidium iodide (PI) dual staining. We observed that 120 μM of BBR induced nearly 29.24% apoptosis at 24 h in Huh-7 cells and 28.03% apoptosis in HepG2 cells (Figure 1B). These results indicated that these two different HCC cell lines responded differently to BBR-induced cytotoxicity, with inhibition of cell growth in a dose- and time-dependent manner.

### 2.2. BBR Inhibits Clonogenic Ability of Huh-7 and HepG2 Cells

To further investigate the influence of BBR, clonogenic abilities of Huh-7 and HepG2 cells were analyzed in a prolonged period of culture time. Since 30 μM of BBR has revealed an inhibitive effect on the viability of both cell lines as shown in Figure 1, this specific concentration was further chosen to carry out the following experiments. After cultivation for 14 days, images of Giemsa staining revealed that 30 μM of BBR could effectively inhibit the clonogenic ability of both Huh-7 and HepG2 cells (Figure 1C).

### 2.3. BBR Causes G0/G1 Phase Cell Cycle Arrest in Huh-7 and HepG2 Cells

**Figure 2:**
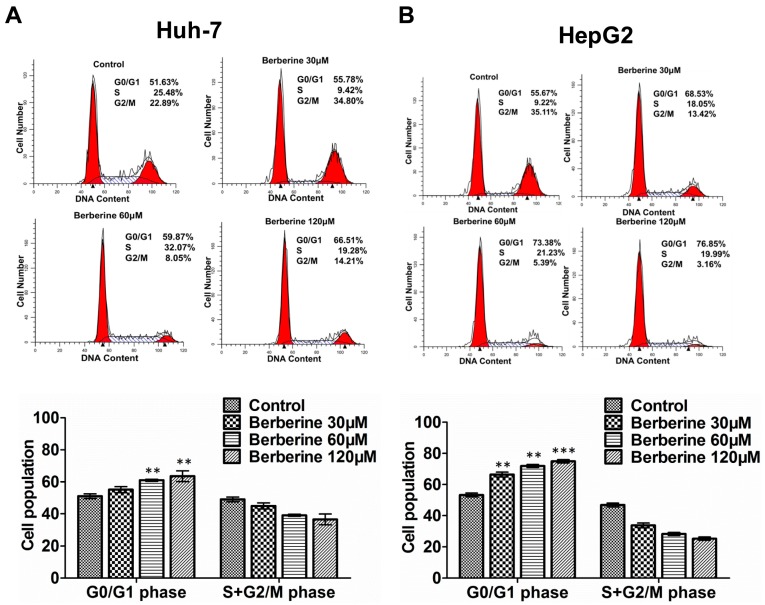
Berberine induces G0/G1 cell cycle arrest in a dose-dependent manner in Huh-7 (**A**) and HepG2 (**B**) cells. Cells were treated with DMSO (control) or berberine (30–120 μM) for 24 h before staining with PI and analyzed by flow cytometer analysis. Distributions of cell cycle are shown in the following bar graph. *** represents *p* < 0.001, and ** represents *p* < 0.01 (60, 120 μM vs. control group). The experiments were carried out for three times.

Since 24 h treatment of BBR could show a satisfactory inhibition effect (Figure 1), following experiments were carried for such duration. After 24 h treatment for different concentrations, PI staining was used to determine the cell cycle profiles of different cells. As shown in Figure 2, BBR could cause G0/G1 phase cell cycle arrest in both Huh-7 and HepG2 cells in a dose-dependent manner. The results also demonstrated that more HepG2 cells were under G0/G1 phase cell cycle arrest than Huh-7 cells after BBR treatment, which was consistent with the changes of viability in Figure 1.

### 2.4. The Induction of BBR-Mediated G0/G1 Phase Cell Cycle Arrest Is Associated with the Regulation of p21^Cip1^, p27^Kip1^ and Skp2 Expression

**Figure 3:**
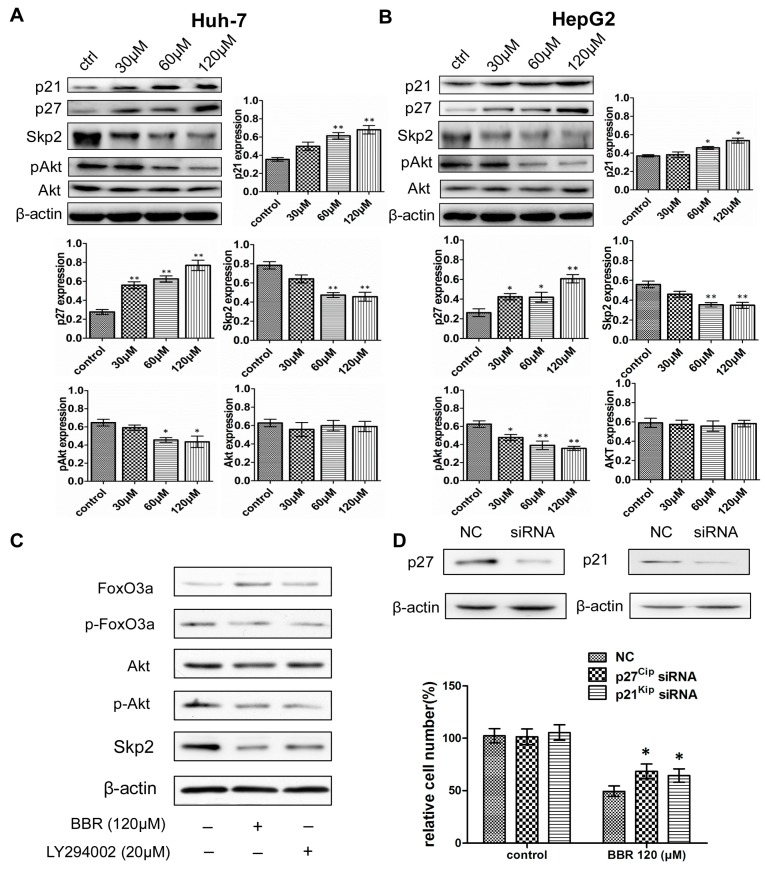
Berberine increases Skp2-mediated p21^Cip1^ and p27^Kip1^ downregulation though abating phosphorylation level of AktHuh-7 cells (**A**) and HepG2 cells (**B**) were treated with DMSO (control) or berberine (30–120 μM) before detecting p21^Cip1^, p27^Kip1^, Skp2, p-Akt (Akt) and β-actin (standard) by western blot. The following bar graphs demonstrate relative expression levels of indicated proteins; (**C**) Huh-7 cells were treated with LY294002 (20 μM), which blocked expression of p-Akt; (**D**) Knockdown of p21^Cip1^ and p27^Kip1^ rescued the suppressive effect of berberine on cell proliferation of Huh-7 cells. CCK8 assay was conducted for cell proliferation. ** represents *p* < 0.01 and * represents *p* < 0.05 (experimental group vs. control group). The experiments were carried out for at least three times.

To investigate the underlying mechanism of BBR-mediated G0/G1 phase cell cycle arrest, expression levels of crucial Cip/Kip family of CDKIs were detected by Western blot. As shown in Figure 3A,B, BBR treatment significantly increased both p21^Cip1^ and p27^Kip1^ in a dose-dependent manner. We also found that siRNA knockdown of p21^Cip1^ and p27^Kip1^ rescued the cell proliferation of Huh-7 cells being treated with 120 μM BBR, confirming their important roles in regulating cell cycle arrest induced by BBR treatment (Figure 3D). Furthermore, since Skp2-containing SCF ubiquitin ligase (SCF^Skp2^) controls the ubiquitylation and degradation of p21^Cip1^ and p27^Kip1^ [21,22,23], which plays a critical role in G1/S transition, the expression levels of Skp2 as well as p21^Cip1^ and p27^Kip1^ were also determined. The results indicated that, together with p21^Cip1^ and p27^Kip1^ accumulation, BBR treatment down regulated Skp2 expression, which induced consequent G0/G1 phase cell cycle arrest.

### 2.5. Activation of Akt Is Involved in BBR-Mediated Skp2 Regulation

As it has been previously reported that Akt could directly phosphorylate Skp2 triggering SCF complex formation and E3 ligase activity [24], we then detected the effects of BBR on the expression of p-Akt and Akt in HCC cells. As shown in Figure 3A,B, the expression level of Akt remained unchanged after BBR treatment, while its activated form (p-Akt) was decreased in a dose-dependent manner. Furthermore, the changed level of p-Akt was more apparent in HepG2 cells, which was consistent with previous results [25]. To investigate the validity of our results, we treated Huh-7 cells with BBR or LY294002, an inhibitor of the phosphatidylinositol 3 kinase (PI3K)/Akt signaling. We found that the protein level of p-Akt was reduced in cells exposed to LY294002, which confirmed its inhibitory effect on the Akt pathway. Moreover, inhibition of the Akt pathway remarkably increased the expression of FoxO3a, while decreasing that of p-FoxO3a and Skp2 (Figure 3C), which were similar to the effected of BBR treatment solely. Therefore, these results suggested that Akt activation is responsible for the role of BBR on cell cycle arrest.

### 2.6. FoxO3a Translocates into Nucleus after BBR Treatment

**Figure 4:**
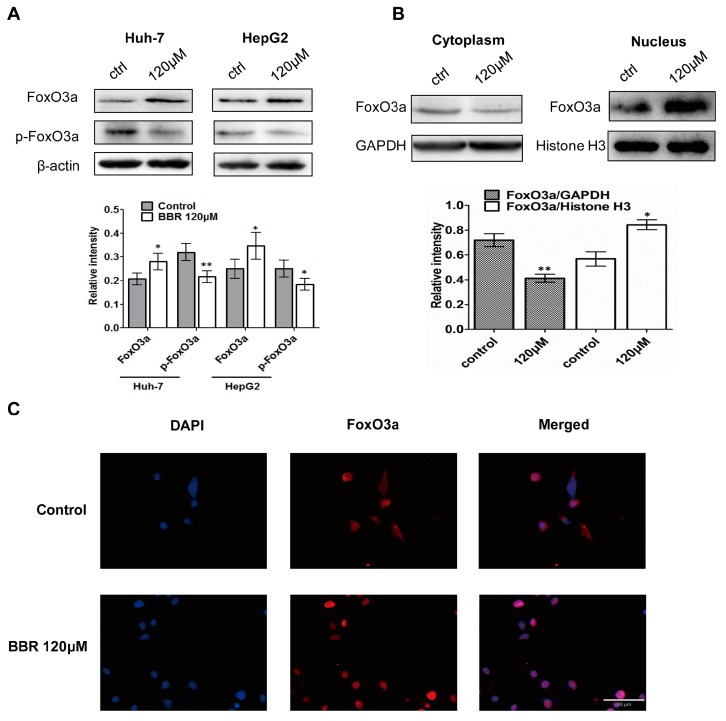
Effects of berberine treatment on FoxO3a and p-FoxO3a expression. (**A**) Huh-7 and HepG2 cells were treated with DMSO or 120 μM berberine for 24 h. Western blot was used to detect changes in the FoxO3a and p-FoxO3a; (**B**) FoxO3a in cytoplasm and nucleus of Huh-7 cells were detected by western blot and normalized by GAPDH and Histone H3 respectively. Relative expression levels are shown in the following bar graph where ** represents *p* < 0.01 and * represents *p* < 0.05 (120 μM group vs. control group.) The experiments were carried out for three times; (**C**) The induction effect of berberine on the nuclear translocation of FoxO3a in Huh-7 cells. Immunofluorescence images stained for anti-FoxO3a (1:100) and DAPI; ** represents *p* < 0.01 and * represents *p* < 0.05 (other experimental groups vs. control group). Scale bar = 90 µm.

Studies have shown that FoxO3a was implicated within cell cycle arrest in different types of cells. Since inactivation of Akt led to decreased phosphorylation of FoxO3a, which can localize in the nucleus to activate transcription target genes such as p21^Cip1^ and p27^Kip1^, and FoxO3a is a negative regulator of Skp2 in a transcription-independent activity [26,27], we detected the phosphorylation level of FoxO3a in Huh-7 and HepG2 cells. In addition to upregulation of FoxO3a, Figure 4A showed that BBR significantly decreased the level of phosphorylated FoxO3a. Next, we determined whether the expression level coincided with changes in localization of FoxO3a after BBR treatment. As shown in Figure 4B, more FoxO3a was translocated from the cytoplasm into the nucleus after treatment with 120 μM BBR in Huh-7 cells, indicating the role of FoxO3a as transcription factor in the expression of p21^Cip1^ and p27^Kip1^. To confirm the nuclear and cytoplasmic fractionation results, immunofluorescence microscopy directly visualized that BBR induced the FoxO3a protein level in the nucleus when compared with untreated Huh-7 cells (Figure 4C).

### 2.7. BBR-Induced G0/G1 Phase Cell Cycle Arrest Is Dependent on FoxO3a Expression

**Figure 5:**
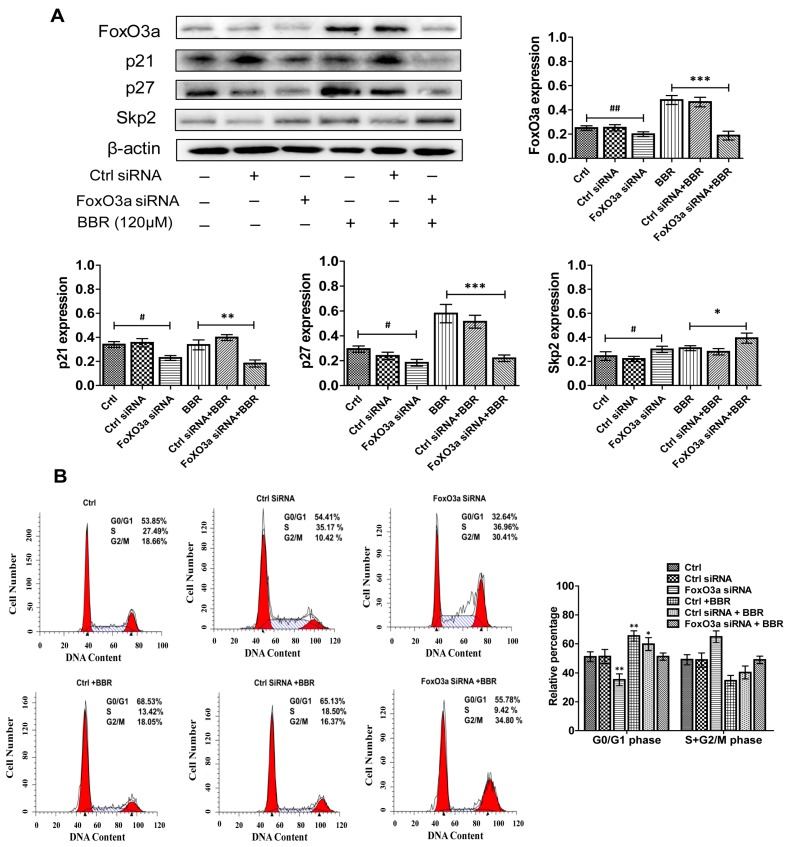
BBR-induced G0/G1 phase cell cycle arrest is attributed to FoxO3a up-regulation in Huh-7 cells. (**A**) Knockdown of FoxO3a induces down-regulation of p21^Cip1^ and p27^Kip1^, up-regulation of Skp2. Huh-7 cells were transfected with control and FoxO3a siRNA with lipofectamine 2000 for 48 h, followed by exposure the cells to berberine (120 μM) for an additional 24 h. The protein levels of FoxO3a, p21^Cip1^, p27^Kip1^ and Skp2 were examined by western blot. Bar graphs demonstrate relative expression levels of indicated proteins. ## represents *p* < 0.01 and # represents *p* < 0.05 (FoxO3a siRNA vs. ctrl group) *** represents *p* < 0.001, ** represents *p* < 0.01 and * represents *p* < 0.05 (FoxO3a siRNA + BBR vs. BBR group); (**B**) Cell cycle analysis for treatment of berberine with FoxO3a siRNA in Huh-7 cells. Silencing FoxO3a attenuated in part the BBR-induced cell cycle arrest in G0/G1 phase. Distributions of cell cycle are shown in the following bar graph. ** represents *p* < 0.01, * represents *p* < 0.05 (other groups vs. control group). The experiments were carried out for three times.

In order to further confirm the role of FoxO3a in the effect of BBR, in this study, Huh-7 cells were incubated with siRNA targeting FoxO3a and the treated with BBR. We found that treatment of the Huh-7 cells with BBR led to an increased expression level of FoxO3a. Moreover, silencing of FoxO3a by siRNA significantly abrogated the BBR-induced p21^Cip1^ and p27^Kip1^ protein expression (Figure 5A). Expectedly, silencing of FoxO3a reversed the effect of BBR on Skp2 protein expression (Figure 5A). Furthermore, it attenuated, in part, the BBR-induced G0/G1 phase cell cycle arrest in Huh-7 cells (Figure 5B). These data indicated that FoxO3a was required in mediating the effect of BBR on p21^Cip1^, p27^Kip1^ and Skp2 protein expression. This also confirmed the important role of FoxO3a in the BBR-induced cell cycle arrest.

## 3. Discussion

Hepatocellular carcinoma (HCC) is an aggressive malignancy with rapid clinical progression. For HCC patients, offering potentially curative treatments is desirable. The investigation of effective anti-cancer drugs is still extensively required in cancer chemotherapy. In our present study, we found that BBR could induce time- and dose-dependent inhibition of the proliferation of Huh-7 and HepG2 cells. Moreover, BBR could effectively abrogate long-term clonogenic survival of Huh-7 and HepG2 cells. As continuous replicative potential is a major cause of neoplastic proliferation and transformation [28], discovering innovative therapies to induce cell cycle arrest could provide new opportunities for treating HCC. Our data showed that 24-h-treatment by BBR could trigger G0/G1 phase cell cycle arrest in Huh-7 and HepG2 cells. Mechanistically, our findings demonstrated that BBR induced cell cycle arrest via reducing the expression level of p-Akt, which in turn diminished Skp2 expression and promoted the expression and nuclear translocation of FoxO3a. The elevated nuclear level of FoxO3a could on one hand, promote the expression of p21^Cip1^ and p27^Kip1^ as a transcription factor, on the other hand, negatively regulate Skp2 expression in a transcription-independent manner [26], leading to accumulation of p27^Kip1^ and p21^Cip1^, and obstruction of G1/S transition. To the best of our knowledge, we have shown for the first time that the Akt/FoxO3a/Skp2 signaling pathway is responsible for BBR-induced proliferative inhibition and cell cycle arrest in Huh-7 and HepG2 HCC cells.

Previous studies have shown that BBR could promote cell apoptosis of HCC cells via a caspase-dependent mitochondrial pathway [10,29], and Fas mediated inhibition of the mammalian target of rapamycin (mTOR) signaling pathway [30]. However, there are no reports covering how BBR induced cell cycle arrest of HCC cells in detail. As we know, the regulatory elements of cell cycle such as cyclins, CDKs, CDKIs, Skp2 and FoxO are key components that respond to mitogenic and survival signals to control the cell cycle, which play an important role in controlling tumor proliferation and cell survival [31,32]. We found that FoxO3a participated vigorously in BBR-induced cell cycle arrest. FoxO3a has been previously described as a tumor suppressor in various tumors, including HCC. As a downstream target of the PI3K/Akt pathway, FoxO3a could mediate cell cycle arrest at the G1/S transition by regulating the transcription of p21^Cip1^ and p27^Kip1^. Many studies demonstrated that Akt activation could directly phosphorylate the tumor suppressor FoxO3a via triggering its nuclear exclusion and subsequent degradation [33]. Therefore, dysregulation of the Akt/FoxO3a signalling axis is a hallmark of oncogenesis, which becomes the potential target of many anti-cancer drugs [34]. Besides, FoxO3a acts as not only a transcription factor, but also a regulator in DNA damage response, which could control damage-induced cell-cycle checkpoints and stimulate DNA repair pathways. This accompanied by the lack of FoxO3a which fails to trigger DNA-repair after DNA damage [35]. In addition, FoxO3a has been reported to be involved in the regulation of cell cycle-related and apoptosis-related gene expression [31,36], and implicated in the oxidative detoxification through regulation of reactive oxygen metabolism by inhibiting mitochondrial gene expression [37]. All these features emphasized the importance of Akt/FoxO3a signaling in BBR-induced cancer cell inhibition.

In the normal cell cycle, the levels of Skp2 remain low in G0/G1 phase and become increased in S phase [38]. Conversely, Skp2 is found to be overexpressed in numerous human cancers, reflecting its crucial role in oncogenesis [39]. The Skp2-SCF complex displays oncogenicity through regulating protein ubiquitination and degradation by its E3 ligase activity, which reveals that Skp2 suppression might be an excellent strategy to inhibit tumorigenesis in tumors. In our research, we found that after BBR treatment, Skp2 level was down-regulated, while p21^Cip1^ and p27^Kip1^ were up-regulated. Consistently, Li Z et al. found that Skp2 participates in cell cycle regulation by degradation of p27^Kip1^ [40]. In addition to the regulation of the post transcriptional level of p27^Kip1^ by Skp2, the ubiquitination and degradation of FoxO3a are also regulated by Skp2 [41]. Moreover, Wu J [26] showed that FoxO3a could directly bind to the Skp2 promoter as a transcriptional repressor, thereby inhibiting the protein expression of Skp2. FoxO3a also displays transcription-independent activity by directly interacting with Skp2 and disrupting Skp2 SCF complex formation, in turn inhibiting Skp2 SCF E3 ligase activity. Therefore, we suspect that FoxO3a/Skp2 is involved in the process of BBR-induced cell cycle arrest in Huh-7 and HepG2 cells. Thus, the results of this study point to an inverse relationship between Skp2 expression and p27^Kip1^ on one hand and, on the other hand, a clear link between Skp2 and FoxO3a activation.

**Figure 6:**
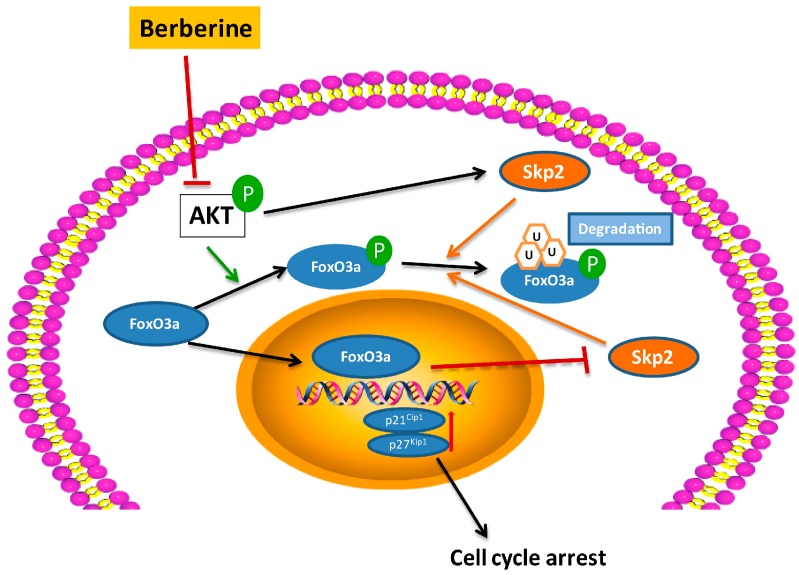
Schematic model of the mechanism underlying the effect of berberine on cell cycle arrest. P: phosphorylation; u: ubiquitylation.

In summary, our results provided evidence, for the first time, that BBR could induce growth inhibition and cell cycle arrest through the Akt/FoxO3a/Skp2 axis in Huh-7 and HepG2 liver cancer cells (Figure 6). This finding suggested a reproducible and reliable anti-cancer effect of BBR, which could improve the understanding of a novel and effective TCM in HCC treatment. Future investigations are needed to explore the upstream involvement of PI3K/Akt in BBR-induced cell cycle arrest, and in vivo studies are required to confirm its effectiveness in the treatment of HCC.

## 4. Materials and Methods

### 4.1. Reagents and Antibodies

BBR was purchased from Sigma-Aldrich (St. Louis, MO, USA) and diluted with Dimethyl Sulfoxide (DMSO, Sigma-Aldrich, MO, USA). Antibodies specific to p21^Cip1^, p27^Kip1^, p-Akt, Akt, GAPDH and Histone H3 were purchased from Cell Signaling Technology (Beverly, MA, USA), antibodies specific to FoxO3a and p-FoxO3a (Ser253) were purchased from HuaBio (Hangzhou, China), antibody specific to Skp2 (p45) and β-actin were purchased from Santa Cruz Biotechnology (Santa Cruz, CA, USA). LY294002 was obtained from Selleck Chemicals (Houston, TX, USA). Human small interfering RNA (siRNA) for FoxO3a and the control siRNA were purchased from Invitrogen. siRNA duplexes sequence against p27 (GTACGAGTGGCAAGAGGUG) and siRNA duplexes sequence against p21 (GCCTTAGTCTCAGTTTGTGTGTCTT), and a non-specific Control (TTCGTAAGAGACCGTGGATCCTGTC) were purchased from GenePharma (Shanghai, China). Lipofectamine 2000 (Invitrogen, Carlsbad, CA, USA) was used for transfection following the manufacturer’s protocols.

### 4.2. Cell Culture

Human hepatoma cell lines Huh-7 and HepG2 cell were purchased from American Type Culture Collection (ATCC, Manassas, VA, USA) and cultured in DMEM (containing 10% fetal bovine serum (FBS, Gibco, Invitrogen, Carlsbad, CA, USA) and 0.1% Penicilin-Streptomycin (Fischer Bioreagents, Pittsburgh, PA, USA)) at 37 °C in a 5% CO_2_ incubator.

### 4.3. Colony Formation Analysis

Huh-7 and HepG2 were seeded in 6-well plate at a density of 100 cells. BBR was added in various concentrations (0 and 30 μM) to the cultured medium that were changed every three days for 14 days. After that colonies were fixed and stained with Giemsa solution. Numbers of individually stained colonies were counted manually.

### 4.4. Cell Viability Analysis

Cell viability was measured by Cell Counting Kit-8 Assay Kit (CCK8, EngreenBiosystem, Beijing, China). Cells were seeded into 96-well plate at a density of 1 × 10^3^ cells per well and were treated with the indicated concentrations (0–120 μM) of BBR for 24 h. CCK8 were add to each well after treatment and incubated for 3 h at 37 °C. The absorbance at 450 nm was detected by ELISA plate reader (Epoch, Biotek, VT, USA). The inhibition rate of BBR was calculated as [(Ac − As)/(Ac − Ab)] × 100% (As, cultured cells with BBR and CCK8; Ac, cultured cell with CCK8 but not BBR; Ab, cultured cells with only CCK8).

### 4.5. Cell Cycle Analysis

Propidium iodide (PI) staining was performed to analyze cell cycles. Briefly, after collecting from 24-well plates, cells were fixed by 70% ethanol for 20 min and washed twice in Phosphate Buffered Solution (PBS). They were stained in 50 uM PI containing 5 μg/mL RNase A for 1 h and analyzed by flow cytometry (FCM) using FACScan (Beckman Coulter, Fullerton, CA, USA).

### 4.6. Phosphatidylserine Externalization Analysis

Following treatment with berberine (30, 60 or 120 µM) for 24 h, cells were harvested and washed with PBS. To quantify the percentage of apoptotic cells, annexin V-FITC and propidium iodide dual staining was performed according to the manufacturer’s protocol (BD Biosciences). Briefly, the cells were resuspended in the binding buffer, and 5 µL Annexin V-FITC staining solution was added and gently mixed. Subsequently, 5 µL propidium iodide (PI) staining solution was added and mixed evenly. Finally, the cells were incubated in the dark for 15 min at room temperature. Samples were analyzed using a FACScan Flow Cytometer. Early apoptotic cells were Annexin V-positive and PI-negative, whereas late apoptotic cells were Annexin V and PI-positive.

### 4.7. Immunoflurescence Staining

Huh-7 cells were first seeded into to dishes overnight, and then, cells were incubated with or without 120 μM berberine for 24 h. Cells were fixed with 4% paraformaldehyde in PBS at 1 h intervals and permeabilized with 0.5% Triton X-100 (Sigma-Aldrich, St. Louis, MO, USA) for 10 min. Cells were blocked with 2% BSA for 30 min. Incubation with primary antibody anti-FoxO3a (diluted 1:100) was done overnight at 4 °C. Immunolabeling was visualized by incubation (3 h at room temperature) with CFTM555 Goat Anti-Rabbit IgG (H + L) (Biotium, Hayward, CA, USA).The nuclei were counterstained with DAPI (Vector Labs, Burlingame, CA, USA). Images were observed and captured with Revolve FL microscope (Echo-labs, San Diego, CA, USA).

### 4.8. Western Blot

After the indicated treatment, cells in 6-well plates were lysed on ice for 15 min by Radio Immunoprecipitation Assay (RIPA) Lysis Buffer (Beyotime Biotechnology, Beijing, China) containing PMSF, protease inhibitor (cOmplete, Roche, Basel, Switzerland), and phosphatase inhibitor (PhosSTOP, Roche, Basel, Switzerland). Cell lysates were centrifuged at 13,000× *g* for 15 min at 4 °C. Proteins in the supernatant were collected and measured by Micro BCA Protein Assay Kit (Thermo Fisher Scientific, Waltham, MA, USA). An equal amount of total protein was separated on 10% SDS-polyacrylamide gel before transferring onto equilibrated PVDF membranes. The membranes were blocked with 5% (*w*/*v*) Bovine serum albumin (BSA, MP Biomedicals, Santa Ana, CA, USA). After that, incubation of specific primary antibodies was carried out overnight at 4 °C. After washing for 3 times with TBST, the membrane was incubated with appropriate secondary antibodies at room temperature for 1 h. Signals were detected by ChemiDoc Touch Imaging System (Bio-Rad, Hercules, CA, USA) using Pierce™ ECL Western Blotting Substrate (Thermo Fischer Scientific, Waltham, MA, USA). Densitometric scanning of the desired band was corrected by β-actin to determine the changed level of specific protein. Each immunoblotting experiment was carried out for at least two times.

### 4.9. Statistical Analysis

SPSS V.12 was used to conduct statistical analysis. Comparisons between groups were assessed by one-way ANOVA and post-hoc analysis (Bonferroni’s test). Statistical values are expressed as mean ± SE. *p*-values < 0.05 were considered as statistically significant.
